# Higher Proportion of Non-1-84 PTH Fragments in Peritoneal Dialysis Patients Compared to Hemodialysis Patients Using Solutions Containing 1.75 mmol/l Calcium

**DOI:** 10.3389/fphys.2018.01643

**Published:** 2018-11-20

**Authors:** Carmen Sánchez-González, Maria Luisa Gonzalez-Casaus, Víctor Lorenzo Sellares, Marta Albalate, José-Vicente Torregrosa, Sebastian Mas, Alberto Ortiz, Mariano Rodriguez, Emilio Gonzalez-Parra

**Affiliations:** ^1^Nefrología, Hospital Universitario La Princesa, Madrid, Spain; ^2^Bioquímica, Hospital Central Gómez Ulla, Madrid, Spain; ^3^Nefrología, Hospital Universitario de Canarias, La Laguna, Spain; ^4^REDINREN, Madrid, Spain; ^5^Nefrología, Hospital Infanta Leonor, Madrid, Spain; ^6^Nefrología y Unidad de Trasplante Renal, Hospital Clinic, Barcelona, Spain; ^7^Unidad de Diálsis IIS Fundación Jiménez Díaz, School of Medicine, Universidad Autónoma de Madrid, Madrid, Spain; ^8^Nefrología y Unidad de Investigación, Hospital Universitario Reina Sofia, Córdoba, Spain

**Keywords:** PTH fragments, non-1-84PTH fragments, 7-84PTH fragments, peritoneal dialysis, low calcium dialysate, low turnover bone disease, 1-84PTH/7-84PTH ratio

## Abstract

**Background:** The prevalence of low- turnover bone disease (LTBD) in peritoneal dialysis (PD) patients is higher than in hemodialysis (HD) patients. LTBD patients may be at risk for vascular calcification, and cardiovascular disease. Current therapy for chronic kidney disease metabolic bone disorders (CKD-MBD) is guided by biochemical parameters, as bone biopsy is not used in routine clinical care.

**Methods:** We assessed intact PTH (iPTH: 1-84PTH plus non-1-84PTH), 1-84PTH, and the 1-84PTH/non-1-84PTH ratio in 129 hemodialysis and 73 PD prevalent patients dialyzed with solutions containing 1.75 mmol/L calcium.

**Results:** Hemodialysis and PD patients presented similar iPTH and tCa values and prevalence of putative LTBD as defined according to KDOQI iPTH cut-off levels or 1-84 PTH levels. However, iCa accounted for a higher percentage of tCa in PD (53%) than in hemodialysis (39%) *p* < 0.001, and the 1-84PTH/non-1-84PTH ratio was lower in PD than in hemodialysis patients (0.44 ± 0.12) vs. (0.60 ± 0.10), *p* < 0.001. The prevalence of putative LTBD when using the coexistence of 1-84PTH/non-1-84PTH ratio < 1.0 and iPTH < 420 pg/m, was higher in PD than in hemodialysis patients (73 vs. 16% respectively, *p* < 0.001). In a multivariate logistic regression analysis, dialysis modality was the main determinant of the 1-84PTH/non-1-84PTH ratio.

**Conclusion:** Solutions containing 1.75 mmol/L calciums are associated to a higher proportion of non-1-84PTH fragments in PD than in HD patients. Different analytical criteria result in widely different estimates of LTBD prevalence, thus impairing the ability of clinicians to optimize therapy for CKD-MBD.

## Introduction

Bone disorders in patients with chronic kidney disease (CKD) encompass high and low turnover bone disease (HTBD and LTBD) (Moe et al., 2006). The prevalence of LTBD appears to be higher in peritoneal dialysis (PD) than in hemodialysis (HD) patients (Rodriguez-Perez et al., 1992; Sherrard et al., 1993; Torres et al., 1995; Couttenye et al., 1997; Sánchez et al., 2000; Levy and Gal-Moscovici, 2008; de Oliveira et al., 2015). Low PTH status and LTBD might be an independent strong risk factor for vascular calcification (VC) (Hutchison et al., 1994; Guérin et al., 2000; London et al., 2004, 2015). Therefore, diagnosis and prevention of LTBD is of great clinical importance in order to identify patients that might benefit from interventions to limit the morbidity and mortality resulting from VC. In parallel, it has been established knowledge that PTH is present in uremic serum in different PTH fragments with variable half-life (Martin et al., 1979). Some of these fragments may even behave as antagonists of the PTH receptor (Langub et al., 2003; Huan et al., 2006). Thus, PTH assays may quantify different peptides with different biological actions and clinical significance (Souberbielle et al., 2006). Second-generation iPTH assays now in widespread clinical use recognize both the full-length molecule (1-84 PTH) and PTH fragments of different sizes missing N-terminal aminoacids, including a 7-84 PTH molecule. 7-84 PTH fragments are found in CKD patients and may behave as partial antagonists of 1-84 PTH, opposing its biological activity. Variants with missing N-terminal aminoacids are generated in the parathyroid glands. Intraglandular aminoterminal degradation is regulated by extracellular ionic calcium (iCa) concentration, which suppresses the release of 1-84 PTH and increases the release of 7-84 PTH fragments from parathyroid cells (Kawata et al., 2005; Friedman and Goodman, 2006). We will use the term non-1-84 PTH throughout the manuscript to refer collectively to these fragments.

There are limited data on the distribution of different PTH fragments according to dialysis modality (Gardham et al., 2010). Recently we have published the first data about such differences (González-Casaus et al., 2014). The aims of the present study were to investigate whether there are any differences in the distribution of circulating PTH fragments in PD vs. HD patients, and additionally we wanted to determine any relationship between PTH fragments and metabolic markers of bone turnover such as the serum Carboxy-terminal telopeptides of collagen type I (βCTx), a marker of bone resorption (Bonde et al., 1995); as well as the possible role of PTH fragments in bone remodeling.

## Patients and Methods

### Patients

This was a cross-sectional study from a historical cohort of 202 Caucasian patients with CKD stage 5 that were dialyzed in two Nephrology centers. Data were collected from all continuous ambulatory peritoneal dialysis CAPD (*n* = 73) and HD (*n* = 129) patients dialyzed with solutions containing 1.75 mmol/L calcium (Table 1). We had blood samples stored at -86°C from these patients. This study was conducted according to the Declaration of Helsinki and approved by the Institutional Review Board and Ethics committee of the Jimenez Díaz Foundation (Ref. 2016/15). Participants were identified by a number and no other identifying material. All included patients gave verbal informed consent.

**Table 1:** Baseline characteristics of the patients.

	Total group	HD group	PD group	*P*-value
	(*n* = 202)	(*n* = 129)	(*n* = 73)	
Age (y)				<0.001
-Mean ± SD	60.17 ± 16.4	64 ± 14.8	52 ± 16.0	
-range	19–82	19–82	22–82	
Sex (% males)	58.4	54.8	63.9	NS
Diabetes (%)	21.4	25.2	14.5	NS
Time on dialysis (y)	2.01 ± 0.82	2.2 ± 0.2	1.63 ± 0.7	<0.001
-Mean ± SD				
Calcitirol (%)	46.7	56.3	29	<0.001


*Clinical characteristics of total study population and the two dialysis subgroups. All patients were being dialyzed with solutions containing the same calcium concentration 1.75 mmol/L calcium. Data expressed as mean ± SD. y, years; HD, hemodialysis; PD, peritoneal dialysis; SD, standard deviation; NS, no significant.*

### Biochemical Parameters

Blood samples were drawn fasting prior to the midweek session in HD and fasting for PD patients, immediately centrifuged, aliquoted and stored at -86°C until analysis. Serum total calcium (tCa) was measured by standard methods in an automated analyzer Cobas Modular Roche. Additionally, ionized calcium (iCa) was quantified in 55 HD patients and all PD patients by ion selective electrode ISE (Rapidpoint 400; Siemens). Serum total 25-hydroxyvitamin D (D2 plus D3) levels were determined by an electrochemiluminiscent assay (ECLIA) in an automated platform/analyzer (LIAISON Vitamin D 25OH Total; DiaSorin Inc) and serum Carboxy-terminal telopeptides of collagen type I (βCTx), a marker of bone resorption (González-Casaus et al., 2014), were measured by ECLIA (CrossLaps, Roche) in an Elecsys 2010 automated system.

The following biochemical parameters were measured simultaneously using a single batch (for automated methods) to minimize analytical variability.

Plasma whole PTH (1-84 PTH, also called bioPTH, determined by a third generation assay) and intact PTH (iPTH: 1-84 plus non-1-84 determined by a second generation assay) were determined simultaneously by an immunoradiometric assay (CA-PTH duo; Scantibodies Laboratory Inc.).

Both assays use an antibody specific for the 39–42 sequence of PTH to immobilize the molecule, but they differ in the second radiolabeled antibody. In the third generation method the second antibody recognizes exclusively the first four aminoacids of the molecule (aminoacids 1–4) to avoid the interference of PTH fragments with larger N-terminal truncations, while in the second generation iPTH assay the second antibody recognizes the 1–34 sequence of PTH. Results were expressed as serum whole 1-84 PTH levels, iPTH levels (1-84 plus non-1-84 PTH) and as a 1-84 PTH/non-1-84 PTH ratio calculated as (1-84 PTH)/iPTH-(1-84 PTH).

According to PTH values, patients were stratified as below, on target, and above PTH values as recommended by KDOQI guidelines (National Kidney Foundation, 2003) and Herberth criteria (Herberth et al., 2010) because both sets of criteria were validated by bone biopsy. As the Allegro iPTH assay (Nichols) (1–84 plus non-1-84 PTH) used to establish the KDOQI reference values was not available, we used the adjustment reported by Souberbielle et al (2006) for iPTH and 1-84 PTH Scantibodies methods to obtain theoretical Allegro iPTH values. Thus, according to KDOQI recommendations, patients with serum iPTH levels < 134 pg/mL (equivalent to < 150 pg/mL Allegro iPTH) were classified as at risk of LTBD, while patients with serum iPTH values > 262 (equivalent to > 300 pg/mL Allegro iPTH) were considered as at risk of HTBD. When whole 1-84 PTH was considered, according to KDOQI recommendations, patients with serum whole 1-84 PTH < 84 pg/mL (equivalent to < 150 pg/mL Allegro iPTH) were classified as at risk of developing LTBD, while patients with serum whole 1-84 PTH > 165 pg/mL (equivalent to > 300 pg/mL Allegro iPTH) were considered as HTBD. In addition, according to Herberth et al. (2010), a 1-84 PTH/ non-1-84 PTH ratio < 1.0 combined with iPTH level < 420 pg/mL was used to diagnose LTBD and a ratio > 1.6 combined with iPTH 340-790 pg/mL for risk of HTBD.

### Statistical Analysis

Standard descriptive statistical analysis was performed and distribution of data was tested using Shapiro-Wilk normality test. Results are expressed as mean or median and 95% confidence interval (CI). Group means were compared using the two-tailed non-paired Student’s *t*-test. Pearson correlation coefficient was used to study the association between quantitative variables. Stepwise multiple regression analysis and partial correlation analysis were used to assess the independent contribution of several variables to bone turnover. All test were two-tailed and the level of significance was set at *p* < 0.05.

## Results

The total study population comprised 118 males and 84 females, with a mean age of 60 years (95% CI: 58–62 years). There were no significant differences in gender distribution or in the presence of diabetes between the two dialysis modalities. Age and time on dialysis were higher in the HD group than in the PD group (Table 1).

Almost half (47%) of participants were treated with active vitamin D (calcitriol), but none received calcimimetics. The percentage of patients treated with calcitriol was higher in the HD group (Table 1). No significant differences were found in cumulative amount of calcium element (g) based on the prescribed treatment of calcium carbonate and calcium acetate during the 12 months prior to the study.

**FIGURE 1:**
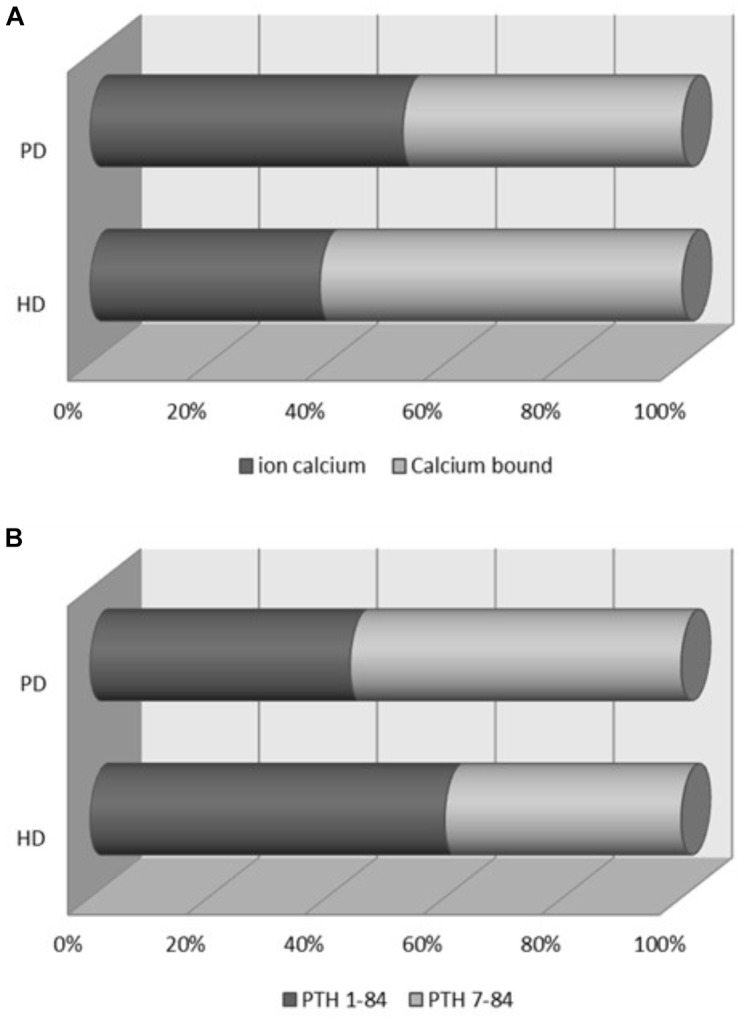
**(A)** Distribution of calcium ion and calcium bound expressed as percentage of total calcium. iCa accounted for 39% of tCa in the HD group and for 53% of tCa in PD patients, *P* < 0.001. **(B)** Distribution of circulating PTH fragments (1-84 PTH and non- 1-84 PTH) expressed as percentage of intact PTH (1-84 PTH plus non-1-84 PTH). Percentage of 1-84 PTH in PD patients (44.0 ± 12.28%) vs. HD patients (60.3 ± 10.82%), *p* < 0.001. HD, hemodialysis; PD, peritoneal dialysis.

There were no differences between dialysis modalities in serum phosphate (mg/dL) [HD: 4.59 (4.4–4.8) vs. PD: 4.73 (4.4–5.1), *p* = 0.294]; tCa (mg/dL) [HD: 9.13 (8.99–9.27) vs. PD: 9.03 (8.8–9.26), *p* > 0.460] or total 25-hydroxyvitamin D [HD: 20.15 (17.25–23.05) vs. PD: 19.46 (17.5–21.4), *p* = 0.546]. However, iCa (mg/dl) levels were lower in the HD [3.52 (3.39–3.61)] vs. PD group [4.76 (4.6–4.84), *p* < 0.001]. In this regard, differences in the distribution of serum calcium were observed; we found a high percentage of iCa in PD patients vs. HD patients (Figure 1A). HD and PD patients presented similar iPTH (pg/ml) values [HD: 204.8 (172–237) vs. PD: 211.2 (139–283), *p* = 0.995]. However, there were differences in the distribution of circulating PTH fragments, which were evident when serum 1-84 PTH levels were expressed as percentage of intact PTH. 1-84 PTH as a percentage of iPTH was significantly lower in PD than in HD (Figure 1B), and 1-84 PTH/non 1-84 PTH ratio much lower in PD as compared to HD patients [PD: 0.88 (0.7–1.1) vs. HD: 1.79 (1.6–2.0), *p* < 0.001]. That finding was corroborated after a multivariate logistic regression analysis including age, gender, diabetes, residual renal function, dialysis vintage, serum phosphate and total serum calcium leves, and vitamin D treatment. iCa showed an inverse correlation with serum 1-84 PTH and 1-84 PTH/non-1-84 PTH ratio in the total study population (Table 2). Serum levels of the bone turnover marker β-CTx correlated with both 1-84 PTH and iPTH in the whole population and also in PD and in HD (Table 2).

**Table 2:** Correlations.

Total group	1-84 PTH	Ipth	1-84PTH/ non 1-84 PTH ratio
iCa	-0.240 (*P* < 0.01)	-0.087 (*P* = 0.336)	-0.636 (*P* < 0.001)
1-84 PTH	–	0.975 (*P* < 0.001)	0.423 (*P* < 0.001)
iPTH	–	–	0.242 (*P* < 0.001)
βCTx	0.441 (*P* < 0.001)	0.399 (*P* < 0.001)	0.280 (*P* < 0.001)
PD group βCTx	0.472 (*P* < 0.001)	0.491 (*P* < 0.001)	0.114 (*P* = 0.411)
HD group βCTx	0.429 (*P* < 0.001)	0.434 (*P* < 0.001)	0.106 (*P* = 0.232)


*Correlations were analyzed by Pearson’s correlation. iCa, ionized calcium; βCTx, carboxy-terminal telopeptides of collagen type I; HD, hemodialysis; PD, peritoneal dialysis; NS, no significant.*

In a multivariate logistic regression analysis including age, dialysis vintage, 1-84 PTH/non-1-84 PTH ratio, vitamin D treatment and dialysis modality, the main determinant of the percentage of calcium present as iCa (iCA/tCa ratio) was the dialysis modality. For this analysis the mean percentage of iCA/tCa ratio (0.47) was used as a cut-off point (constant: 3.91; Estimate: 0.001, 95% CI: 0.000-0.012, *P* < 0.001). Similarly, the main determinant of LTBD (defined as the coexistence of a 1-84 PTH/non-1-84 PTH ratio < 1.0 and iPTH < 420 pg/mL), was iCa concentration (Table 3).

**Table 3:** Independent contributing factors to low bone turnover disease, defined by a 1-84 PTH/ non 1-84 PTH ratio ≤ 1 and iPTH < 420 pg/mL, in the multiple regression analysis.

Coefficient	iCa	iCa^∗^ (a)	iCa^∗^ (a & v)
Constant	10.67	7.19	6.93
Estimate	-9.85	-8.68	-8.75
Exp (B) (95% CI)	0.000 (0.000–0.002)	0.000 (0.000–0.006)	0.000 (0.000–0.006)
*P*-value	<0.001	<0.001	<0.001


*iCa^∗^ (a), ionized calcium corrected by age; iCa^∗^ (a & v), ionized calcium corrected by age and dialysis vintage; iCa, ionized calcium.*

**FIGURE 2:**
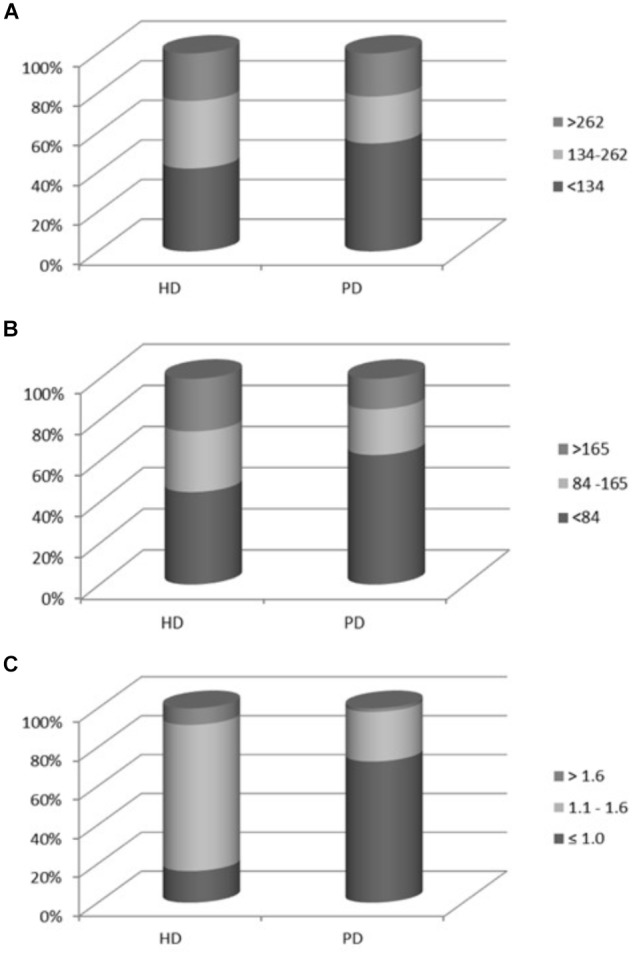
Diagnosis of bone turnover in dialysis according to: **(A)** KDOQI recommended cut-off levels for *iPTH Scantibodies assay* normalized according to Soubervielle to obtain the equivalent Allegro iPTH values. [*HTBD*: iPTH Scantibodies > 262 pg/ml = Allegro iPTH > 300 pg/ml; *NORMAL*: iPTH Scantibodies 134–262 pg/ml = Allegro iPTH 300-150 pg/ml; *LTBD*: iPTH Scantibodies < 134 pg/ml = Allegro iPTH < 150 pg/ml]. **(B)** *Whole 1-84 PTH assay* normalized according to Soubervielle to obtain the equivalent Allegro iPTH values. [*HTBD*: whole 1-84 PTH > 165 pg/ml = Allegro iPTH > 300 pg/ml; *NORMAL*: whole 1-84 PTH: 84–165 pg/ml = Allegro iPTH 300–150 pg/ml; *LTBD:* 1-84 PTH < 84 pg/ml = Allegro iPTH < 150 pg/ml]. **(C)** *1-84 PTH/* *non-1-84 PTH ratio* < 1.0 combined with iPTH level < 420 pg/mL for developing LTBD and a ratio > 1.6 combined with iPTH 340–790 pg/mL for risk of HTBD. Prevalence of LTBD in PD (72.7%) vs. HD patients (16.3%), *P* < 0.0001. HD, hemodialysis; PD, peritoneal dialysis, HTBD, high turnover bone disease; LTBD, low turnover bone disease.

There were no significant differences in the distribution of HD or PD patients into LTBD or HTBD when bone turnover was defined according to KDOQI recommended cut-off levels for iPTH, either when iPTH was normalized to Allegro iPTH values (Figure 2A) or when whole 1-84 PTH was normalized to Allegro iPTH values (Figure 2B). However, defining LTBD or HTBD according to 1-84 PTH/ non-1-84 PTH ratio for the diagnosis of bone turnover in dialysis led to a significantly higher prevalence of LTBD in PD than in HD patients (Figure 2C). In accordance with these findings, serum β-CTx (pmol/L) was significantly lower in the PD group [1181 (946–1393)] vs. HD patients [2084 (1633- 2238). *p* < 0.001], suggesting also a lower bone turnover activity in PD patients.

## Discussion

This study investigated differences in serum levels of different PTH fragments between PD and HD patients using solutions containing 1.75 mmol/l calcium. 1-84 PTH, as a percentage of iPTH, and 1-84 PTH/non-1-84 PTH ratio were lower in the PD than in the HD group. The association between 1-84 PTH/non-1-84 PTH ratio and dialysis modality was corroborated by multivariate regression models. In addition, this study the concordance between different cut-off points to biochemically suspect LTBD according to guidelines or individual author’s suggestions. The main finding is that there is little concordance between guideline-based cut-off points for iPTH or 1-84PTH and the proposal by Herbeth et al. based on a study of HD using a combination of iPTH and 1-84 PTH/non-1-84 PTH ratio (Herberth et al., 2010). A higher percentage of PD patients and a lower proportion of HD patients were diagnosed of suspected LTBD when 1-84 PTH/ non-1-84 PTH ratio were applied for the diagnosis of bone turnover in dialysis. This view would be further supported by the lower levels of serum β-CTx in PD patients. By contrast iPTH alone did not disclose differences in putative LTBD between HD and PD. Furthermore, these findings are consistent with the previous observation that high serum iPTH levels, assessed by second-generation assays, reflecting the sum of potentially opposing effects of 1-84 PTH and its fragments, do not correlate with histomorphometric data in patients with LTBD (Torres et al., 1995; Wang et al., 1995; Sánchez et al., 2000; Barreto et al., 2008), more frequently observed in PD patients, as well as in patients with HTBD (Herberth et al., 2009; Garrett et al., 2013). The recommendations of different guidelines on the target PTH levels are not uniform between the different guidelines an even within some guidelines; the recommended range has changed through years. In the past the aim was to maintain patients between 150 and 300 pg/ml; more recently the upper limit was increased to 6 times the upper normal (Kidney Disease, 2009). While the present study represents routine clinical practice and, thus, lacks bone biopsies to confirm LTBD, it does raise a series of issues regarding currently used cut-off points and their trustworthiness to guide therapy for secondary hyperparathyroidism. Matters are complicated by the commercial availability of different PTH assays with wide inter-method variability (Gardham et al., 2010). The individual PTH values obtained in the same sample using different assays may potentially point to opposing diagnostic and therapeutic attitudes (Souberbielle et al., 2006).

Although controversy exists, it has been proposed that assessment of the different PTH fragments may provide information on bone turnover in patients undergoing dialysis (Monier-Faugere et al., 2001; Herberth et al., 2010). According to this view, the higher percentage of PD patients with evidence of LTBD when 1-84 PTH/7-84 PTH ratio was applied for the diagnosis of bone turnover in dialysis than when iPTH criteria was applied, might represent a true higher incidence of LTBD and would point to the inaccuracy of KDOQI suggested thresholds for higher risk of LTBD. However, the lack of bone biopsy precludes confirmation of this hypothesis in the present cohort.

Renal replacement therapy is associated with net influx of Ca to the patient when dialysate calcium concentration is higher than serum calcium. Furthermore, serum iCa level in PD patients, as a consequence of its continuous nature, may be higher than in HD patients (Kurz et al., 1995) as illustrated by the present report, especially when 1.75 mmol/L calcium PD fluids are used (Hutchison et al., 1992; Weinreich et al., 1995; Sanchez et al., 2004; Haris et al., 2006; Yamamoto et al., 2008; Soroka et al., 2011; Yee-Moon Wang, 2014). Changes in serum iCa concentration are sensed by the CaSR that signals to regulate PTH secretion and regulate processing of PTH to yield different fragments (Habener et al., 1975; D’Amour et al., 1992; Brown and MacLeod, 2001; D’Amour, 2002). High serum iCa levels favor non-1-84 PTH fragments secretion over whole 1-84 PTH decreasing the 1-84 PTH/non-1-84 ratio. Thus, in our PD cohort higher serum iCa favored by the 1.75 mmol/L calcium PD fluid led to higher secretion of non–1-84 PTH fragments such as 7-84 PTH thus promoting LBTD (Slatopolsky et al., 2000; Divieti et al., 2002; Ok et al., 2016). Other factors than PTH level might regulate osteoblast function in renal osteodystrophy in PD such as high serum glucose, cytokines and local bone growth factors that decrease bone formation making LTBD.

Despite the uncertainty, given the available evidence, including the fact that the current cohort of PD patients used 1.75 mmol/L calcium PD fluid, a known risk factor for LTBD (Merle et al., 2016; Ok et al., 2016), we would concur that the combination of iPTH and 1-84 PTH/non-1-84 PTH ratio might be useful to diagnose LBTD also in PD patients and may be more sensitive that the iPTH currently used for this purpose.

Several weaknesses should be recognized. We did not perform bone biopsies in our population as they are not part of the current standard of care. The absence of this gold standard impedes to draw definitive conclusions about the true prevalence of LBTD in our PD patients and the relative accuracy of the combination of iPTH and 1-84 PTH/non-1-84 PTH ratio to non-invasively diagnose LBTD.

In conclusion, these findings cast doubt on the reliability of available cut-off points for PTH values that are in use or have been proposed to guide therapy for secondary hyperparathyroidism in dialysis patients. While the lack of bone biopsies does not allow to validate any of the cut-off vales as accurate, the higher iCa and lower β-CTx levels in the present cohort of PD patients are consistent with the hypothesis that conventional KDOQI cut-off points do underestimate the prevalence of LTBD in PD patients. Given that there is no biopsy, we can only conclude that there is increased likelihood of LTBD in PD patients based on biomarker using Herberth/KDOQI criteria.

## Author Contributions

CS-G, MA, VS, J-VT, and MR performed the clinical research. MG-C performed the analysis. MR wrote the manuscript. SM, AO, and EG-P corrected and edited the manuscript.

## Conflict of Interest Statement

The authors declare that the research was conducted in the absence of any commercial or financial relationships that could be construed as a potential conflict of interest.
